# Perceived truth of statements and simulated social media postings: an experimental investigation of source credibility, repeated exposure, and presentation format

**DOI:** 10.1186/s41235-020-00251-4

**Published:** 2020-11-11

**Authors:** Lena Nadarevic, Rolf Reber, Anne Josephine Helmecke, Dilara Köse

**Affiliations:** 1 Department of Psychology, School of Social Sciences, University of Mannheim, 68131 Mannheim, Germany; 2 Department of Psychology, University of Oslo, 0373 Oslo, Norway

**Keywords:** Fake news, Social media, Source credibility, Truth effect, Truthiness

## Abstract

To better understand the spread of fake news in the Internet age, it is important to uncover the variables that influence the perceived truth of information. Although previous research identified several reliable predictors of truth judgments—such as source credibility, repeated information exposure, and presentation format—little is known about their simultaneous effects. In a series of four experiments, we investigated how the abovementioned factors jointly affect the perceived truth of statements (Experiments 1 and 2) and simulated social media postings (Experiments 3 and 4). Experiment 1 explored the role of source credibility (high vs. low vs. no source information) and presentation format (with vs. without a picture). In Experiments 2 and 3, we additionally manipulated repeated exposure (yes vs. no). Finally, Experiment 4 examined the role of source credibility (high vs. low) and type of repetition (congruent vs. incongruent vs. no repetition) in further detail. In sum, we found no effect of presentation format on truth judgments, but strong, additive effects of source credibility and repetition. Truth judgments were higher for information presented by credible sources than non-credible sources and information without sources. Moreover, congruent (i.e., verbatim) repetition increased perceived truth whereas semantically incongruent repetition decreased perceived truth, irrespectively of the source. Our findings show that people do not rely on a single judgment cue when evaluating a statement’s truth but take source credibility and their meta-cognitive feelings into account.

## Significance statement

With the ongoing digitalization and the frequent use of social media in everyday life, the amount of information passed on has increased substantially. Consequently, people encounter more news than they can properly evaluate. To better understand the mechanisms that promote people’s belief in (fake) news, we examined whether the perceived veracity of statements and news headlines, respectively, depend on 1) source credibility, 2) repeated exposure, and 3) presentation format (with vs. without a picture). We found that the perceived truth of statements was higher when allegedly presented by expert sources or reliable news sources than by laypersons or dubious news sources. Regardless of a statement’s source, however, previous exposure to the statement increased its perceived truth. Likewise, previous exposure to a textually similar but semantically incongruent statement decreased perceived truth. Pictures accompanying the statements did not have any effects. Taken together our findings show that people consider source credibility when forming truth judgments. However, they also tend to believe information they have encountered before and to distrust information that deviates from previously encountered information. The order of information processing thus plays a central role in people’s evaluations of (fake) news. Moreover, cross-experimental comparisons suggest that making source information more salient could be an effective means to diminish the effect of previous statement exposure on judged truth.

## Introduction

With the increasing use of social media (e.g., Twitter and Facebook) as a means to obtain current news, the amount of information disseminated has increased substantially. Unfortunately, not only does the amount of true information increase, but also the amount of false information such as insufficiently verified reports, rumors, and fake news (i.e., intentionally misleading or fabricated news). Although the phenomenon of fake news existed long before the invention of the Internet (Burkhardt 2017; Lazer et al. 2018), with the extensive use of social media platforms its spread has reached a whole new level. For instance, a large-scale analysis of 126,000 Twitter stories revealed an even faster and broader diffusion of false than true information (Vosoughi et al. 2018). Although users’ sharing behavior via social media is not yet well understood, it is reasonable to assume that people are more likely to post and share information they believe in (Lazer et al. 2018; but see Pennycook and Rand 2020). In order to better understand why and when people believe in (fake) news, it is of utmost importance to study the cognitive processes underlying human information processing, to identify variables predicting the perceived truth of information (Brashier and Marsh 2020), and to explore how they act in combination. In this regard, the present work experimentally investigated whether source credibility, repeated exposure, and presentation format predict the perceived truth of statements and simulated social media postings.

### Fake news and determinants of truth judgments

According to Lazer et al. (2018), fake news is “fabricated information that mimics news media content in form, but not in organizational process or intent. Fake-news outlets, in turn, lack the news media’s editorial norms and processes for ensuring the accuracy and credibility of information” (p. 1094). Given this definition of fake news, an important indicator of the veracity of encountered information is its source. In fact, source credibility is a strong predictor of the persuasiveness of information (see Pornpitakpan 2004, for a review). Yet if news sources are taken into account when evaluating information, how is it possible that people fall for fake news? One reason is that source credibility is only one of several judgment cues that people use to evaluate the veracity of information. For instance, Pennycook et al. (2018) demonstrated that repeated statement exposure increases people’s belief in (fake) news. In their study, true and false news headlines appeared in a typical social media layout, i.e., the headlines appeared with a source reference and a picture. Headlines seen for the second time were perceived to be more accurate than headlines seen for the first time. However, the authors did not assess source effects and picture effects on participants’ judgments.

In fact, to our knowledge, no study has ever investigated simultaneous effects of source credibility, statement repetition, and accompanying pictures on truth judgments. Hence, it is unclear to what extent these three variables influence people’s belief in (fake) news when they come together—which is typically the case in the context of social media. The present work aims at closing this research gap. To this end, we will start by introducing central theories and findings on (1) source credibility, (2) statement repetition, and (3) accompanying pictures as determinants of perceived truth. In this context, we will of course also present research on joint effects, if available.

#### Source credibility

Source credibility is one of several possible source characteristics. Although it is often studied as a unidimensional construct, source credibility contains the sub-facets expertise and trustworthiness (Pornpitakpan 2004). Typically, information provided by credible sources has a larger impact on recipients’ beliefs, attitudes, and behaviors than information provided by non-credible sources (for a meta-analysis, see Wilson and Sherrell 1993). For instance, Heinbach et al. (2018) presented participants with a made-up news article on superfoods supposedly coming from a high-credibility or low-credibility German news website (sueddeutsche.de vs. bild.de). As expected, participants in the high-credibility source condition showed a stronger attitude change towards superfoods in line with the article’s arguments than participants in the low-credibility source condition. A meta-analysis by Kumkale et al. (2010) reported effect sizes of *d* = 0.35 (fixed effects) and *d* = 0.42 (random effects) for source credibility effects on attitude change, if participants attitudes were assessed directly after message exposure. However, the authors also identified several moderator variables such as people’s prior knowledge, the strength of prior attitudes, and the delay of attitude assessment.

Research on source credibility is historically linked to dual-processing models of persuasion (Chaiken et al. 1989; Petty and Cacioppo 1986). These models assume that recipients use source credibility as a heuristic cue for an argument’s strength, if they lack the ability or motivation to elaborate on the argument’s quality. Due to these historical roots, most studies that examined source credibility used complex stimuli such as news articles, stories, or reports to investigate persuasive effects. However, there are also some studies that addressed source credibility effects on the perceived truth of short propositions (e.g., Begg et al. 1992; Henkel and Mattson 2011; Law 1998). For instance, Law (1998) presented participants with short marketing claims (e.g., *British Airways has flown the greatest number of transcontinental passengers*) supposedly coming from a trustworthy source (e.g., a consumer report) or from an untrustworthy source (e.g., a TV commercial). After a delay of 15 min, these claims were presented for a second time together with new, similar claims. This time, however, all claims appeared without source information and participants had to judge each statement’s truth. Truth judgments were higher when a statement had been presented by a trustworthy compared to an untrustworthy source. However, this was only the case if participants still remembered the source. Else, participants provided higher truth ratings for the claims from untrustworthy sources than for the new claims. This finding points to another determinant of judged truth: repetition.

#### Statement repetition

Long before the invention of the Internet and social media, a survey by Allport and Lepkin (1945) discovered that people are more likely to believe in rumors they had heard before than in unfamiliar rumors. The first experimental demonstration of this *truth effect* was provided by Hasher et al. (1977), who examined the perceived truth of repeatedly presented trivia statements. Since then the effect has been replicated numerous times (see Unkelbach et al. 2019, for a review). A standard truth effect experiment consists of (at least) two phases: In the exposure phase, participants are exposed to true and false statements and instructed to process these statements in a certain way (for different processing tasks, see Hawkins and Hoch 1992; Nadarevic and Erdfelder 2014). In the judgment phase, some or all of these statements are presented a second time together with several new statements. This time, participants have to judge the truth of each statement (e.g., on a Likert-scale ranging from definitely false to definitely true). Typically, mean truth judgments for the repeated statements turn out to be higher than mean truth judgments for the new ones. A meta-analysis by Dechêne et al. (2010) revealed a medium effect size for this *between-items* truth effect (*d* ≊ 0.50). The effect size increases drastically, however, if participants are not informed about the factual number of true and false statements presented in the exposure phase (Jalbert 2018). Thus, most laboratory studies presumably underestimate the real-world impact of repeated statement exposure.

The most common theoretical explanation for the truth effect is the fluency account (Begg et al. 1992; Reber and Schwarz 1999; Unkelbach 2007). It assumes that repetition enhances a statement’s processing fluency and that people rely on this metacognitive experience of processing ease when judging the truth of statements. According to the *referential theory* (Unkelbach and Rom 2017), the experience of fluency depends on a statement’s fit with a recipient’s semantic network. The more references the statement shares with the network and the more coherently the statement fits into the network, the more fluently it is processed at re-exposure and the more likely it will be judged as true. Hence, the referential theory highlights the role of conceptual rather than perceptual fluency. In fact, although there are demonstrations of perceptual fluency effects on truth judgments (e.g., Reber and Schwarz 1999; see also Graf et al. 2018), they are much smaller and less robust than conceptual fluency effects on perceived truth (Parks and Toth 2006; Silva et al. 2016; Vogel et al. 2020).

Unkelbach and Greifeneder (2018) claimed that people’s truth judgments do not depend on a single judgment cue, but on several ecologically valid cues, if available. Moreover, they reasoned that people integrate declarative cues (e.g., knowledge) and experiential cues (e.g., fluency) when making truth judgments.^1^ The authors tested this prediction by presenting statements together with declarative advice from one of three fictitious persons (e.g., *GALI says: This statement is true*) in the judgment phase of a truth effect experiment. Importantly, advice validities differed between the three fictitious persons (e.g., 50%, 60%, and 70%) and were explicitly communicated. As hypothesized, participants integrated statement repetition and advice validity into their truth judgments, i.e., both variables affected truth judgments in an additive manner. Yet, does this finding replicate if source information instead of explicit advice is provided at the time of judgment? To our knowledge, no experiment so far has addressed this question. Although some studies investigated joint effects of statement repetition and source credibility, these truth effect studies only provided source information in the initial exposure phase, not in the truth judgments phase (e.g., Begg et al. 1992; Henkel and Mattson 2011; Law 1998). This means that the findings strongly depended on participants’ source memory. Therefore, the contributions of source credibility and repeated exposure on the judged truth of statements in general and social media postings in particular are far from clear. Moreover, many social media postings have an additional feature in common that may also influence truth judgments: accompanying pictures.

#### Accompanying pictures

Compared to traditional print news articles, social media news postings tend to contain pictures. Some studies show that pictures presented in scientific texts, such as graphs presented with medical information or brain images in neuroscience articles, improve the rated persuasiveness of a text (Tal and Wansink 2016) and judgments of whether the scientific reasoning behind a claim makes sense (McCabe and Castel 2008). A large-scale replication of the study of McCabe and Castel (2008), in contrast, could find little to no evidence for a persuasive effect of brain images (Michael et al. 2013).

Research on an effect called *truthiness* (Newman et al. 2012) suggests that even non-probative pictures—i.e., pictures that lack any diagnostic power with regard to the veracity of the information presented—may increase people’s truth judgments for thematically related statements. For instance, Newman et al. (2012) presented participants with trivia statements (e.g., *Macadamia nuts are in the same evolutionary family as peaches*) that either appeared with or without a picture. The pictures displayed the grammatical subject of the statement (e.g., Macadamia nuts) but did not provide any information about the statement’s veracity. Participants judged the statements with an accompanying picture as true more often than the statements presented without a picture.

Even though the truthiness effect has been replicated several times (e.g., Fenn et al. 2013; Newman et al. 2015, 2020), its magnitude is typically quite small. For instance, a mini meta-analysis by Newman et al. (2020) showed an average effect size of *d* = 0.23. Moreover, there are several contextual constraints of the effect. First, pictures have to be semantically related to the respective statements in order to produce truthiness (Newman et al. 2015). Second, Cardwell et al. (2016) found a moderating effect of statement valence. The authors replicated truthiness for positive, but not for negative claims. Finally, similar to the truth effect, the empirical demonstration of truthiness requires a within subject design, i.e., participants have to evaluate both statements with *and* statements without pictures. Based on this finding, Newman et al. (2020) reasoned that statements that appear with semantically related pictures feel easier to process compared to statements without such pictures. More precisely, the authors argued that the pictures enhance the conceptual fluency of statements by activating relevant concepts in people’s semantic networks. Hence, presumably the truth effect and truthiness share the same underlying cognitive mechanism. What is unclear so far is whether statement repetition and accompanying pictures have additive effects on perceived truth or whether the apparently stronger fluency effect (repetition) overrides the weaker one (pictures). To our knowledge, no study has ever investigated the truthiness effect and the truth effect in the same study. Likewise, we are not aware of a study that investigated joint effects of source credibility and truthiness.

Beyond assessing which of these three effects is strongest, we were also interested in interaction effects. For example, individuals use metacognitive cues when no direct information can be accessed (Haddock et al. 1999). If so, we would expect less pronounced effects of metacognitive cues through repetition and non-probative pictures when direct information, such as when an expert source underpins a claim than when a less trustworthy source or no source accompanies the message. However, as all manipulated variables are peripheral cues, it is also possible that they just act in an additive manner.

### The present research

Although source credibility, statement repetition, and accompanying pictures demonstrably influence truth judgments when studied in isolation, it is unclear (1) whether these variables jointly contribute to the perceived truth of statements and social media news headlines, (2) which of these variables has the largest impact on rated truth, and (3) whether they affect truth judgments in an additive or multiplicative manner. The following four experiments aimed to answer these questions and to gain a better understanding of the mechanisms that contribute to people’s belief in (fake) news.

We defined a minimum sample size of *N* = 60 for each experiment. A power analysis with G*Power (Faul et al. 2007) indicated that given this sample size, an α-level of 0.05, and an estimated repeated-measures correlation of *ρ* = 0.20, the power of finding moderate-sized effects (*f* = 0.25) in our experiments was larger than 0.85 for the tested main effects. Moreover, our G*Power analyses indicated a power larger than 0.80 for the interactions of source credibility and pictures or source credibility and repetition, respectively.^2^ We will report all data exclusions (if any), all manipulations, and all measures in the methods sections of the individual experiments. The materials and the data of all experiments are publicly available online at the Open Science Framework (OSF; https://osf.io/bnqgs/).

## Experiment 1

Tweets on Twitter and postings on Facebook come from a variety of sources such as private individuals, public institutions, companies, or news agencies. It is reasonable to assume that people evaluate a statement such as “*Ibuprofen prevents severe courses of Covid-19*” differently if it stems from an institutional source (e.g., the Ministry of Health) than if it stems from an unknown layperson (e.g., the engineer Jorun Rolfsen) as the two sources differ in expertise with regard to the statement’s subject. The previously introduced findings on source credibility suggest that expert sources should increase the perceived truth of a statement. The expected effect of lay sources, in contrast, is less clear. Do people consider statements from lay sources to be less likely true than statements of unknown origin? Andrews and Rapp (2014) observed that people are less likely to accept information from low-credibility sources than information from sources of unknown credibility. However, it is unclear whether lay sources fall into the category of low-credibility sources or sources of unknown credibility. In the latter case, it is even conceivable that any source reference, even one to a lay source, increases perceived truth relative to a no-source condition. Experiment 1 investigated this issue by presenting statements together with the name of an expert source, an unknown lay source, or without source information. Importantly, we did not provide participants with any background information on the alleged sources, in order to keep the study as naturalistic as possible. On social media platforms such as Facebook, for example, the only information about the origin of a statement or posting, respectively, is the name of the source without further details on source characteristics. Moreover, because information on social media not only comes with a source reference but often with an accompanying picture as well, Experiment 1 aimed to explore joint effects of source credibility (expert, layperson, no source) and presentation format (with picture, without picture) on the perceived truth of statements. In line with the truthiness effect, we predicted that thematically related, but non-probative pictures increase the perceived truth of the presented statements.

### Methods

#### Participants

Eighty-eight students (67 female, 21 male) from the University of Oslo, Norway, completed the experiment online. We excluded one participant because she had provided uniform judgments to all statements. The final sample thus consisted of 87 participants, most of whom belonged to the age group of 16–25 years (*n* = 73). The others were in the age groups of 26–35 years (*n* = 11), 36–45 years (*n* = 1), and 46–55 years (*n* = 2). All participants were proficient in Norwegian and received course credit for participation.

#### Design

The experimental design was a 3 (source: expert vs. layperson vs. no source) × 2 (picture: yes vs. no) within-subjects design. Mean truth ratings (1 = “definitely false” to 6 = “definitely true”) served as the dependent variable.

#### Materials

We selected 60 statements on social issues such as education, health, and politics that were collected from news and political sources from the Internet. In line with Henkel and Mattson (2011), we did not check the veracity of the statements. In fact, statements from the Internet with uncertain veracity correspond to the material we typically encounter in everyday life and thus strengthen the ecological validity of the study. For each of the 60 statements (e.g., *UiO is Norway's oldest institution for research and higher education, with 27,000 students and 6,000 employees*), we selected an expert source (e.g., University of Oslo) and a lay source (e.g., Restorer Ingvild Fosse). Expert sources were names of institutional sources and scientific journals, respectively, that we selected based on their thematic fit to the respective statement. Lay sources were fictitious names that were provided together with a position or activity that was clearly unrelated to the content of the statement. Non-probative pictures were taken from the Internet and matched the general topic of the statement but did not provide any relevant information that could determine the truth status of the statement.

The 60 statements were counterbalanced across all cells of the experimental design by means of six participant groups so that each statement appeared in a different condition in each group. Of the 60 statements presented in each group, 20 were displayed with an expert source, 20 were displayed with a lay source, and 20 appeared without source information. Moreover, half of the statements (i.e., ten in each source condition) were presented together with an associated but non-probative picture whereas the other half was presented without a picture. The assignment of statement conditions to the six counterbalancing groups is outlined in the supplementary materials on OSF.^3^

#### Procedure

Participants were recruited through SONA, an online participant pool system. After giving informed consent, participants received instructions to judge the statements according to their truth and were informed that the experimenters were interested in their spontaneous responses. Then, the 60 statements were successively presented in random order on the screen and participants provided their ratings on a six-point scale (1 = “definitely false”; 6 = “definitely true”). If a source was displayed, it appeared below the statement (e.g., source: University of Oslo). Pictures (if present) also appeared below the respective statement or below the source. After the 60 statements, the participants were thanked, debriefed, and received credit.

### Results

In this and the following experiments, all statistical tests refer to an α-level of 0.05. Moreover, in case of violation of the sphericity assumption (as indicated by Mauchly’s test) degrees of freedom are Greenhouse–Geisser corrected.

#### Analysis of variance

**Fig. 1:**
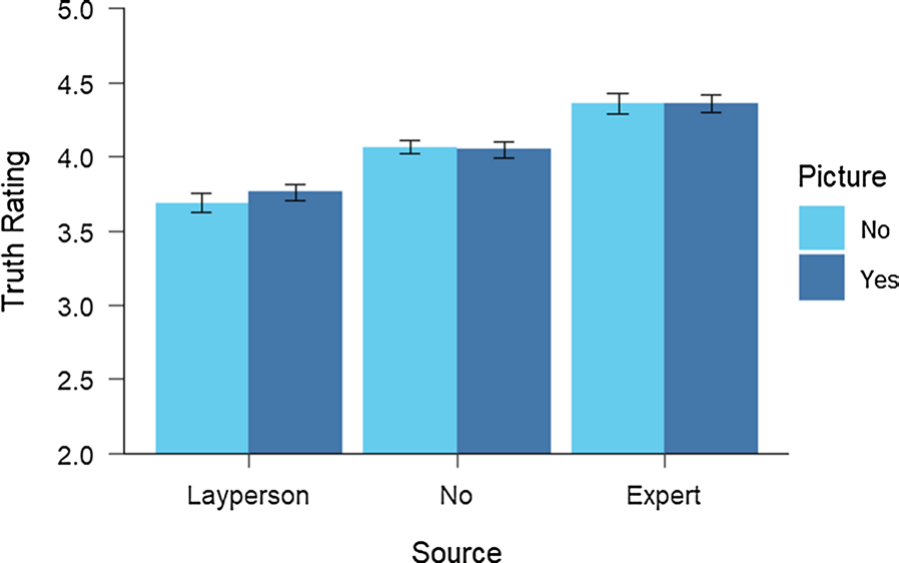
Mean truth ratings in Experiment 1 as a function of source and picture conditions. Error bars represent standard error of the means

A 3 (source: expert vs. layperson vs. no source) × 2 (picture: yes vs. no) repeated-measures ANOVA was run with mean truth judgments as the dependent variable. As expected, participants’ truth judgments varied depending on a statement’s source, *F*(1.69, 145.21) = 50.86, *p* < 0.001, ${\eta }_{\mathrm{p}}^{2}$ = 0.37. Simple contrasts to the no-source condition revealed that truth judgments were higher in the expert-source condition (*M* = 4.36, *SD* = 0.50) than in the no-source condition (*M* = 4.06, *SD* = 0.37), *F*(1, 86) = 27.59, *p* < 0.001, ${\eta }_{\mathrm{p}}^{2}$ = 0.24, and lower in the lay-source condition (*M* = 3.73, *SD* = 0.48) than in the no-source condition, *F*(1, 86) = 37.77, *p* < 0.001, ${\eta }_{\mathrm{p}}^{2}$ = 0.31. In contrast, pictures did not influence truth judgments (with picture: *M* = 4.06; *SD* = 0.34; without picture: *M* = 4.04; *SD* = 0.38), *F* < 1, and there was also no source by picture interaction, *F* < 1. Figure 1 depicts the descriptive results.

#### Linear mixed model

Due to the incomplete counterbalancing of one statement, we additionally analyzed our data with a linear mixed model that accounts for random variance in participants and items. We used the R-packages lme4 (Bates et al. 2014) and lmerTest (Kuznetsova et al. 2017) for this analysis. The tested model included the fixed factors source condition and picture condition and the interaction between the two. Furthermore, the model included random intercepts for participants and statements. In line with the ANOVA findings, truth judgments were significantly affected by source, *F*(2, 5065.3) = 133.81, *p* < 0.001, but not by picture, *F*(1, 5066) = 1.10, *p* = 0.295. Again, there was no interaction between the two factors, *F* < 1.

### Discussion

The results of Experiment 1 show that people consider source information when forming truth judgments. Importantly, however, they do not merely consider the availability (or absence) of source information but take the credibility of the source into account. Specifically, we found that expert sources increased the perceived truth of statements whereas lay sources decreased the perceived truth compared to a control condition without source information. Surprisingly, we did not find an effect of non-probative pictures on truth judgments. We will elaborate on this finding in further detail in Experiment 2.

## Experiment 2

According to Unkelbach and Greifeneder (2018), people integrate declarative judgment cues (e.g., source credibility) and experiential judgment cues (e.g., fluency) into their truth judgments. In Experiment 1, however, participants exclusively relied on source credibility as a cue. In contrast, non-probative pictures did not affect truth judgments, even though they presumably enhance processing fluency. Because Experiment 1 is the first study that has investigated joint effects of source credibility and non-probative pictures on judged truth, we can only speculate why participants solely focused on source information (*does the statement come from an expert, a layperson, or is the source unknown?*) while neglecting the pictures. Possibly, the integration of declarative cues and experiential cues requires specific conditions. For instance, the strength of people’s experiential feelings could play a central role. Because repetition is one of the strongest and most popular fluency manipulations, we included statement repetition as an additional factor in Experiment 2. In fact, Unkelbach and Greifeneder (2018) had observed additive effects of repetition and declarative advice on truth judgments. Thus, including repetition as a further factor allowed us to test the replicability and generalizability of their findings. Moreover, as stated earlier, investigating joint effects of statement repetition, source credibility, and presentation format is also important for applied reasons, because the three variables typically co-occur in the context of social media.

### Methods

#### Participants

Ninety-one students (71 female, 16 male, 4 not specified) from the University of Oslo, Norway, completed the experiment online. Of these, *n* = 75 belonged to the age group of 16–25 years, *n* = 10 to the age group of 26–35 years, *n* = 2 to the age group of 36–45 years, and *n* = 2 to the age group of 46–55 years. Two participants did not indicate their age. Participants were proficient in Norwegian and received course credit for participation.

#### Design

The experimental design was a 3 (source: expert vs. layperson vs. no source) × 2 (repetition: yes vs. no) × 2 (picture: yes vs. no) within-subjects design. Mean truth ratings served as the dependent variable.

#### Materials

The materials were the same as in Experiment 1. In order to counterbalance repeated statements versus new statements across participants, we divided the 60 statements into two sets of 30 statements each. Half of the participants received the first set in the exposure phase, the other half received the second set. Participants received all 60 statements in the judgment phase. Half of these statements were old (i.e., repeated) and half of them were new for each participant. We counterbalanced source types and picture conditions across repetition conditions.^4^

#### Procedure

The procedure of Experiment 2 was similar to Experiment 1, except for the following changes. First, there was an exposure phase and a judgment phase. Participants had to judge the interestingness of 30 statements on a scale from 1 (“little interesting”) to 6 (“very interesting”) in the exposure phase and the truth of all 60 statements in the judgment phase. In the exposure phase, we presented only the statements, without sources and pictures. Second, participants completed a 10-item non-verbal filler task between the exposure phase and the judgment phase in order to minimize the impact of short-term memory for the last statements (see Postman and Phillips 1965). The filler task consisted of two-row matrices with the digits 1 to 6 displayed in the upper row and the symbols < , X, O, + , –, and & displayed below. Each item consisted of an initial matrix (identical across trials) and a rearranged matrix, in which one symbol was missing. The participants’ task was to identify the missing symbol.

### Results

#### Analysis of variance

**Fig. 2:**
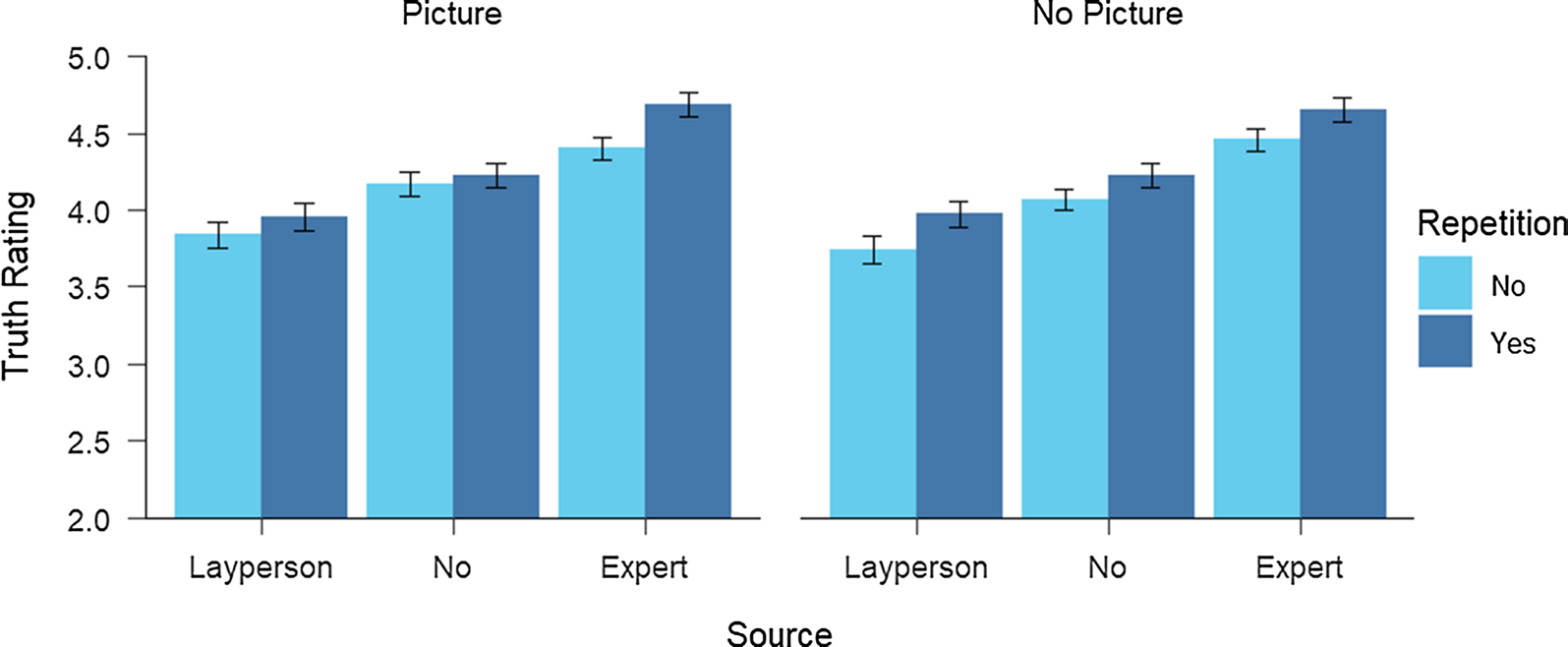
Mean truth ratings in Experiment 2 as a function of picture, source, and repetition conditions. Error bars represent standard error of the means

A 3 (source: expert vs. layperson vs. no source) × 2 (repetition: yes vs. no) × 2 (picture: yes vs. no) repeated-measures ANOVA was run with mean truth judgments as the dependent variable. As in Experiment 1, source had a strong impact on participants’ truth judgments, *F*(1.64, 147.17) = 47.98, *p* < 0.001, ${\eta }_{\mathrm{p}}^{2}$ = 0.35. The pattern of this source effect was also identical to Experiment 1. Simple contrasts to the no-source condition revealed that truth judgments were higher in the expert-source condition (*M* = 4.55, *SD* = 0.56) than in the no-source condition (*M* = 4.17, *SD* = 0.53), *F*(1, 90) = 37.36, *p* < 0.001, ${\eta }_{\mathrm{p}}^{2}$ = 0.29, and lower in the lay-source condition (*M* = 3.88, *SD* = 0.69) than in the no-source condition, *F*(1, 90) = 25.39, *p* < 0.001, ${\eta }_{\mathrm{p}}^{2}$ = 0.22. We also found truth judgments to be higher for repeated statements (*M* = 4.28, *SD* = 0.56) than for new statements (*M* = 4.11, *SD* = 0.46), *F*(1, 90) = 12.90, *p* < 0.001, ${\eta }_{\mathrm{p}}^{2}$ = 0.13, thus replicating the truth effect. In contrast, truth judgments were again unaffected by non-probative pictures (with picture: *M* = 4.21, *SD* = 0.50; without picture: *M* = 4.18, *SD* = 0.47), *F* < 1, and there were no interactions between the three factors, *F*s ≤ 1.59, *p*s ≥ 0.207, ${\eta }_{\mathrm{p}}^{2}$s ≤ 0.02. This means that the effects of source and repetition were additive (see Fig. 2).

#### Linear mixed model

We additionally analyzed our data with a linear mixed model because, as in Experiment 1, one statement was unbalanced across experimental conditions. The tested model included the fixed factors source, repetition, and picture and all possible interactions between these factors as well as random intercepts for participants and statements. In line with the ANOVA findings, truth judgments were significantly affected by source, *F*(2, 5300.3) = 159.66, *p* < 0.001, and repetition, *F*(1, 5300.7) = 30.19, *p* < 0.001, but not by picture, *F* < 1. Again, there were no interactions between any factors, *F*s ≤ 1.67, *p*s ≥ 0.189.

### Discussion

In Experiment 2, we found strong, additive effects of source credibility and statement repetition on judged truth. Expert sources increased the perceived truth of statements whereas lay sources decreased the perceived truth compared to a control condition without source information. Moreover, truth judgments were higher for repeated compared to new statements. These findings are consistent with the general pattern of results reported by Unkelbach and Greifeneder (2018) and thus provide evidence for their replicability and generalizability. Accordingly, our findings support the assumption that people integrate declarative cues (e.g., source information) *and* experiential cues (e.g., a statement’s fluency) when forming truth judgments. Unlike repetition, however, non-probative pictures did not show any influences on truth judgments. That means, as in Experiment 1, the truthiness effect did not replicate. We will come back to this point in a later section.

## Experiment 3

Experiment 3 tested the generalizability of the previous findings using a different sample population (German instead of Norwegian participants) and a different set of materials. The experiment was similar to Experiment 2, except that statements were framed as news headlines and the presentation layout was similar to the one on the Facebook social media platform. We implemented these changes in order to simulate a social media news context. Within this framework source credibility was manipulated by either presenting a statement together with the name and logo of a trustworthy, real news source (real source), together with the name and logo of an untrustworthy, made-up news source (fake source), or without any source information (no source). As in Experiment 2, we also varied repeated exposure to statements (yes vs. no) and the presentation format of the statements (with picture vs. without picture). Despite the mentioned changes, we expected to replicate the findings of Experiment 2.

### Methods

#### Participants

Participants were recruited at the University of Mannheim, Germany. Sixty-four participants (51 female, 13 male) completed the experiment. The participants’ age was in the range of 19–57 years (*M* = 23.5, *SD* = 6.2). The majority of participants (*n* = 57) were native German speakers, the others indicated having very good (*n* = 5) or good (*n* = 2) German skills.^5^ Participants received course credit (*n* = 42) or volunteered for a piece of cake.

#### Design

The experimental design was the same as in Experiment 2.

#### Materials

We collected 120 statements, most of them from the Internet. As in the previous experiments, and in line with Henkel and Mattson (2011), we did not check the statements’ veracity. We rephrased the statements so that they reflect the typical style of a news headline. That is, all statements included a buzzword or short introduction followed by the actual message (e.g., *New contraceptive for men: Researchers have developed a contraceptive gel that has been successfully tested in monkeys*). Additionally, we assembled 32 real German news sources and made up 16 names for news sources that factually do not exist (i.e., *fake sources*).

The 120 statements and 48 sources were evaluated in a pre-test (*N* = 25) in which participants judged the truth of each statement (1 = “definitely false”; 6 = “definitely true”), indicated for each source whether it was familiar (yes vs. no) and rated the trustworthiness of each source (1 = “very untrustworthy”; 9 = “very trustworthy”). Based on the pre-test, we selected 60 statements (truth ratings: 3.0 < *M* < 4.0, SD < 2.0). Moreover, we selected ten real news sources that were judged as familiar by at least 20 of the 25 pre-testers and ten fake sources that were judged as familiar by no more than 1 of the 25 pre-testers. Mean trustworthiness ratings were above the midpoint of the scale (*M* > 5.0) for the ten real sources and below (*M* < 5.0) for the ten fake sources.

We also conducted a second pre-test (*N* = 10) in which the source names were presented together with logos (official logos for real sources vs. made-up logos for fake sources). Presenting the sources together with logos helped participants to differentiate between familiar sources and unfamiliar sources. For this reason, and for the sake of ecological validity, we decided to present the sources together with their logos in the experiment.

We then searched for two thematically associated, non-probative pictures for each of the 60 selected statements. To decide which of the two pictures was better suited, another five pre-testers evaluated the thematic fit of each picture to the corresponding statement (1 = “does not fit at all”; 5 = “fits very well”) and judged whether the picture provided information about the veracity of the statement (1 = “no, not at all”; 5 = “yes, absolutely”). We only selected pictures that—according to the pre-testers—provided low information about veracity (*M* < 3.0) and therefore were non-probative. If both pictures met this criterion, we chose the picture with the higher thematic-fit rating.

Based on the selected materials, we created two stimulus sets, each consisting of 30 statements that were comparable with regard to their mean pre-tested truth ratings (Set A: *M* = 3.58; Set B: *M* = 3.54; both *SD*s = 0.27). The two sets served to counterbalance which statements were repeatedly presented and which statements were not. Next, we created pairs of real and fake news sources (e.g., CNN and KKN). These source pairs were then assigned to three statements within each set based on their thematic fit to the real news source. For example, statements on an economic topic were assigned to a source that typically reports on economic affairs. Each statement was counterbalanced across all cells of the experimental design according to a Latin square.

#### Procedure

**Fig. 3:**
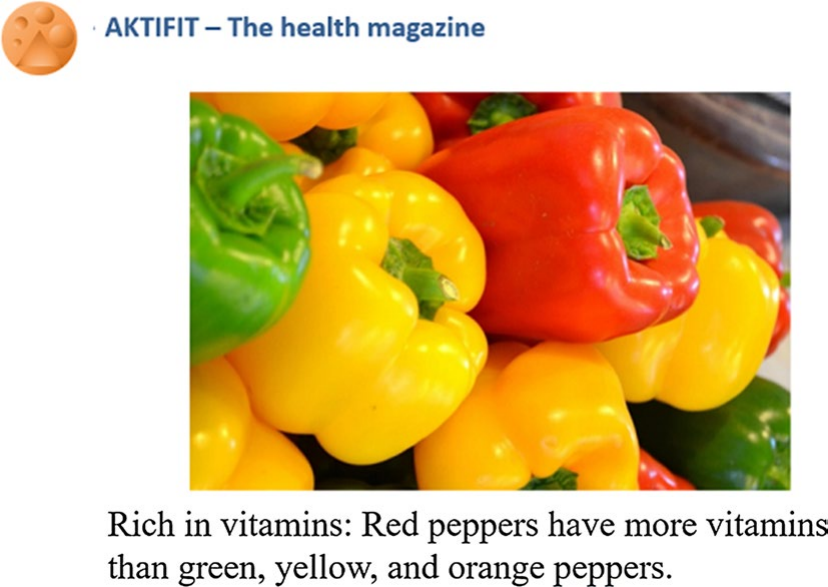
Exemplary statement in the fake-source condition and picture condition. Note that in the original study the sources and statements were presented in German. The picture displayed is under public domain license (CC0) and is from https://pixnio.com/de/pflanzen/gemuse/pfeffer/paprika-paprika-pfeffer-gemuse

The procedure of Experiment 3 was similar to Experiment 2, except for the following changes. First, this time the judgment phase directly followed the exposure phase. Second, we told participants at the start of the judgment phase that the statements would appear together with corresponding pictures and source information, if available. In fact, the assignment of sources and pictures was completely under experimental control. This deception served to unravel source effects from statement effects. If a source was present, it was displayed in the upper left corner of the simulated news posting (see Fig. 3). Finally, the experiment involved a source judgment phase in which the 20 sources (including their logos) were successively displayed in random order. For each source, participants had to indicate whether they had been familiar with the source prior to the experiment (yes vs. no) and to rate the trustworthiness of the source (1 = “very untrustworthy”; 9 = “very trustworthy”).

### Results

#### Manipulation check

On average, participants judged 95% of the real sources and 2% of the fake sources as familiar. Moreover, as expected and in line with the pre-tests, they rated the real sources as significantly more trustworthy (*M* = 6.96, *SD* = 1.06) than the fake ones (*M* = 3.52, *SD* = 1.10), *t*(63) = 20.82, *p* < 0.001, *d*_*z*_ = 2.60. This indicates that participants were able to distinguish between credible and dubious news sources.

#### Analysis of variance

**Fig. 4:**
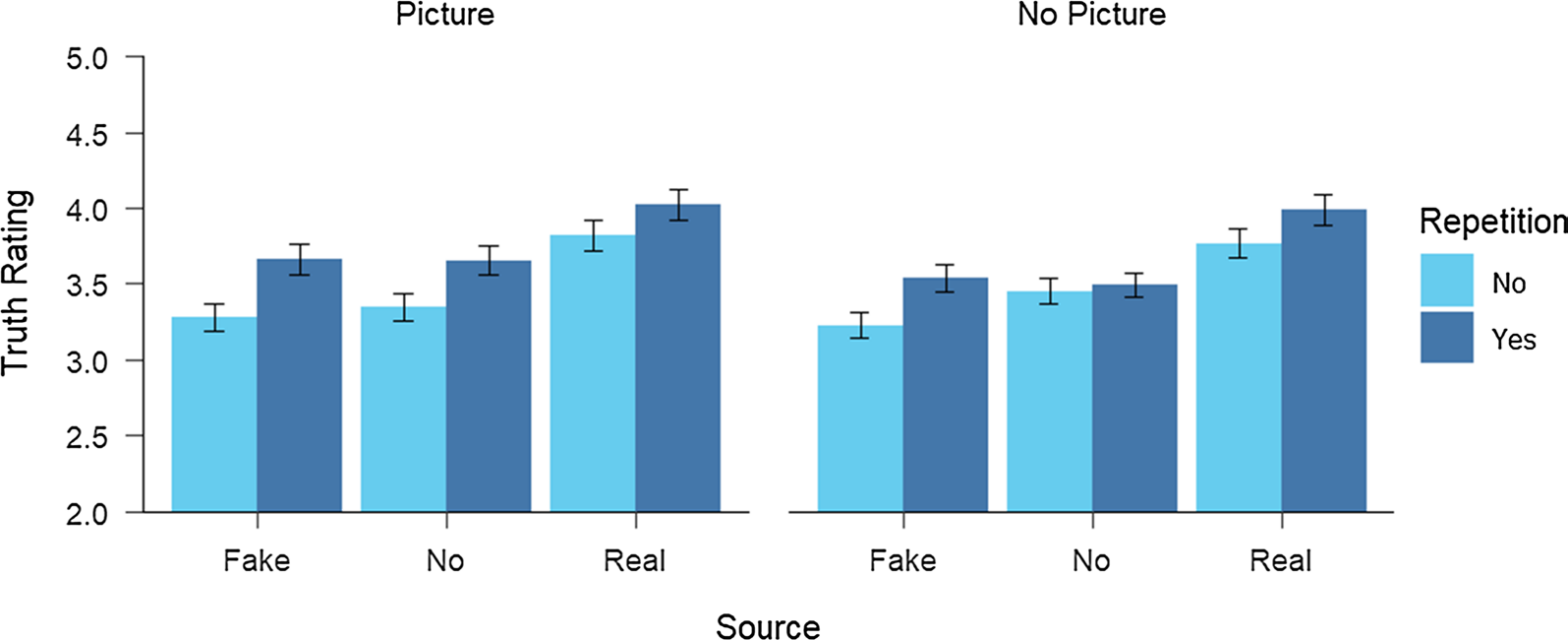
Mean truth ratings in Experiment 3 as a function of picture, source, and repetition conditions. Error bars represent standard error of the means

A 3 (source: real vs. fake vs. no source) × 2 (repetition: yes vs. no) × 2 (picture: yes vs. no) repeated-measures ANOVA was run with mean truth judgments as the dependent variable. In line with Experiment 2, source had the strongest impact on the participants’ truth judgments, *F*(1.66, 104.72) = 26.20, *p* < 0.001, ${\eta }_{\mathrm{p}}^{2}$ = 0.29. Simple contrasts showed that truth judgments were higher in the real-source condition (*M* = 3.90, *SD* = 0.62) compared to the no-source condition (*M* = 3.49, *SD* = 0.46), *F*(1, 63) = 24.91, *p* < 0.001, ${\eta }_{\mathrm{p}}^{2}$ = 0.28, but did not differ between the latter and the fake-source condition (*M* = 3.43, *SD* = 0.46), *F*(1, 63) = 1.20, *p* = 0.277, ${\eta }_{\mathrm{p}}^{2}$ = 0.02. We also replicated the truth effect obtained in Experiment 2, i.e., truth judgments were higher for repeated statements (*M* = 3.73, *SD* = 0.52) than for new statements (*M* = 3.48, *SD* = 0.43), *F*(1, 63) = 15.00, *p* < 0.001, ${\eta }_{\mathrm{p}}^{2}$ = 0.19. Once again, there was no effect of non-probative pictures on truth judgments (with picture: *M* = 3.63, *SD* = 0.48; without picture: *M* = 3.58, *SD* = 0.40), *F*(1, 63) = 1.32, *p* = 0.255, ${\eta }_{\mathrm{p}}^{2}$ = 0.02, and there were no interactions, *F*s ≤ 2.17, *p*s ≥ 0.129, ${\eta }_{\mathrm{p}}^{2}$s ≤ 0.03. Hence, as in Experiment 2, the source effect and the truth effect were additive (see Fig. 4).

#### Linear mixed model

We also explored whether the above findings would replicate when predicting truth judgments not as a function of real versus fake sources but as a function of participants’ source trustworthiness ratings. We did so by running a linear mixed-model analysis. The model included the fixed factors rated source trustworthiness (as a continuous variable), statement repetition (yes vs. no), picture presentation (yes vs. no) and all possible interactions between these factors. Moreover, the model included random intercepts for participants and statements. Truth judgments from the no-source condition were discarded from the following analysis. In line with the ANOVA findings, truth judgments were significantly affected by perceived source trustworthiness, *F*(1, 2534.7) = 133.75, *p* < 0.001, and by statement repetition, *F*(1, 2430.4) = 32.95, *p* < 0.001. Moreover, again, there was no effect of non-probative pictures *F*(1, 2431.7) = 1.68, *p* = 0.196, and no interactions between any factors, *F*s < 1.

### Discussion

Despite a different sample population, a modified presentation layout, and other materials, the results of Experiment 3 were essentially the same as in Experiment 2. Again, we found strong, additive effects of source credibility and repetition on participants’ truth ratings, but no picture effect and no interactions. The only notable difference was in the pattern of the source effect. In contrast to the lay sources, fake sources did not decrease the perceived truth of statements compared to the no-source condition. This is remarkable, particularly in light of the low explicit credibility ratings for the fake sources.

## Experiment 4

Different news sources often report on the same information. For this reason, it is likely to come across the same statement repeatedly when skimming social media news headlines. Experiments 2 and 3 demonstrated that previous exposure increases the perceived truth of a statement regardless of its source. However, what happens when encountering a news headline that is inconsistent with or even contradictory to another headline seen before (e.g., *Ibuprofen promotes severe courses of Covid-19* vs. *Ibuprofen prevents severe courses of Covid-19*)? Previous studies suggest that people tend to disbelieve statements that are semantically incongruent with previously seen or heard statements (Bacon 1979; Garcia-Marques et al. 2015; Silva et al. 2017; Unkelbach and Rom 2017). This *illusion of falseness*—i.e., lower truth ratings for incongruently repeated statements compared to new statements—is particularly likely if the time interval between the processing of an initial statement and an incongruent one is rather short (Garcia-Marques et al. 2015). But it is an open question whether the illusion of falseness also replicates in a simulated social media news context that includes source information.

According to the *Discrepancy-Induced Source Comprehension model* (D-ISC model, Braasch and Bråten 2017), attentiveness to sources increases if people come across semantically incongruent information. To our knowledge, however, no study has yet investigated whether source credibility affects truth judgments differently in the case of incongruently repeated statements than in the case of congruently (i.e., verbatim) repeated statements or new statements. For this reason, the aim of Experiment 4 was to investigate joint effects of type of repetition (congruent repetition, incongruent repetition, no repetition) and source credibility (real source, fake source) on the perceived truth of alleged social media headlines. Please note that for the sake of ecological validity, we no longer included a no-source condition. Moreover, because pictures had not affected truth judgments in our previous experiments, we also dropped this experimental factor. Instead, all statements appeared together with a picture and a source reference in the truth judgment phase to keep the study as naturalistic as possible.

### Methods

#### Participants

Participants were recruited at the University of Mannheim. Eighty participants (66 female, 14 male) completed the experiment. The participants’ age was in the range of 18–50 years (*M* = 22.0, *SD* = 4.4). The majority of participants (*n* = 75) were native German speakers, the others indicated to have very good (*n* = 4) or good (*n* = 1) German skills. Participants received course credit (*n* = 54) or volunteered for a piece of cake.

#### Design

The experimental design was a 2 (source: real vs. fake) × 3 (repetition: congruent vs. incongruent vs. no repetition) within-subjects design. Mean truth ratings served as the dependent variable.

#### Materials

We constructed 120 pairs of statements. Each pair consisted of two statements that were semantically incongruent with one another but differed in only one term (e.g., *Export economy: Turkey/Italy produces 70% of all hazelnuts worldwide*). Some of the statements were adapted from Experiment 3, other were newly created based on contents found on the Internet. As in the previous experiment, all statements had a typical news-headline format. Although we were no longer interested in the effect of pictures, we decided to present each statement with a picture, because this is the typical presentation format of social media news headlines. For this reason, we selected a non-probative picture for each statement-pair that fitted the content of both statements. We submitted all statements to a pre-test (*N* = 40). The pre-testers’ task was to judge the truth (1 = “definitely false”; 6 = “definitely true”) of the statements, which were displayed together with the selected pictures. Importantly, pre-testers never judged two statements of the same pair as these appeared in separate between-subject conditions. Based on the pre-test, we selected 54 statement-pairs (truth ratings of the individual statements: 3.0 < *M* < 4.0).

The sources were the same as in Experiment 3. However, at the time of preparing Experiment 4, the trustworthiness of the German news source SPIEGEL had fallen into disrepute due to the so-called *Relotius scandal* (Fichtner 2018). For this reason, we decided to omit the source “SPIEGEL Online” and its fake counterpart. Therefore, we only used nine of the ten source pairs (real–fake pairings) of Experiment 3, which we assigned to the 54 statement pairs based on their thematic fit. Statements of the same pair were assigned to two different sets. Each statement was then counterbalanced across all cells of the experimental design according to a Latin square.

#### Procedure

The procedure was the same as in Experiment 3, except for the following changes. In the exposure phase, participants judged the interestingness of 36 statements. Eighteen of these statements reappeared as verbatim repetitions in the judgment phase, i.e., they were repeated congruently. The other 18 statements contained a semantic modification compared to the exposure phase, i.e., they were repeated incongruently. In addition, the judgment phase involved 18 new statements. The participants’ task was to rate the truth of the 54 statements, each of which was presented together with a picture and a source. In the final source judgment phase, participants again provided binary familiarity judgments as well as trustworthiness ratings for each of the 18 sources in random order.

### Results

#### Manipulation check

On average, participants judged 93% of the real sources and 3% of the fake sources as familiar. Moreover, as in the pre-tests and in Experiment 3, they rated the real sources as significantly more trustworthy (*M* = 6.92, *SD* = 0.83) than the fake ones (*M* = 4.02, *SD* = 0.97), *t*(79) = 20.21, *p* < 0.001, *d*_*z*_ = 2.26. This indicates that participants were able to distinguish between credible and dubious news sources.

#### Analysis of variance

**Fig. 5:**
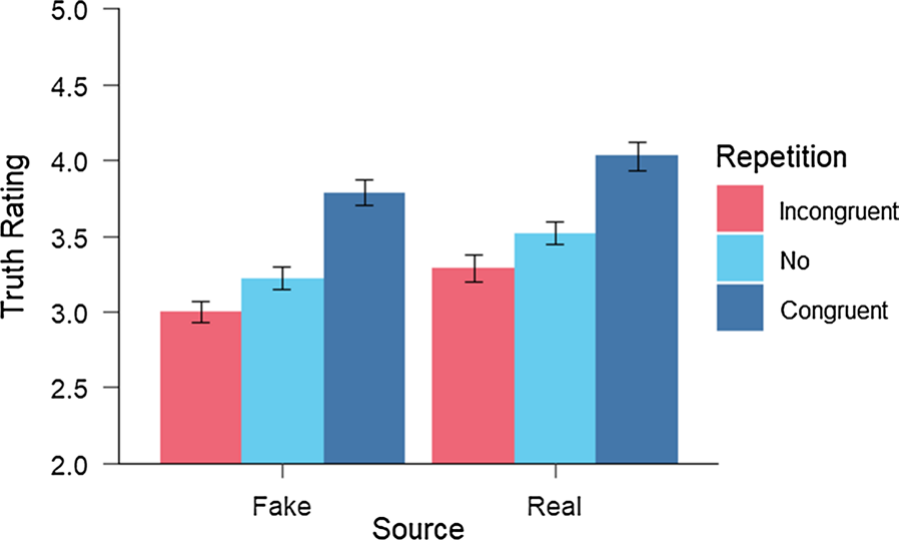
Mean truth ratings in Experiment 4 as a function of source condition and repetition condition. Error bars represent standard error of the means

A 2 (source: real vs. fake) × 3 (repetition: congruent, incongruent, no repetition) repeated-measures ANOVA was run with mean truth judgments as the dependent variable. As in the previous experiments, the alleged source of a statement had a strong effect on participants’ truth judgments, *F*(1, 79) = 17.42, *p* < 0.001, ${\eta }_{\mathrm{p}}^{2}$ = 0.18. Truth judgments in the real-source condition (*M* = 3.61, *SD* = 0.57) were higher than truth judgments in the fake-source condition (*M* = 3.33, *SD* = 0.39). Likewise, statement repetition affected truth judgments, *F*(1.64, 129.78) = 35.93, *p* < 0.001, ${\eta }_{\mathrm{p}}^{2}$ = 0.31. Simple contrasts confirmed the expected pattern of statement repetition. Compared to non-repeated statements (*M* = 3.37, *SD* = 0.54) congruent statement repetition increased truth judgments (*M* = 3.91, *SD* = 0.71), *F*(1, 79) = 32.92, *p* < 0.001, ${\eta }_{\mathrm{p}}^{2}$ = 0.29, whereas incongruent repetition decreased truth judgments (*M* = 3.14, *SD* = 0.60), *F*(1, 79) = 10.25, *p* = 0.002, ${\eta }_{\mathrm{p}}^{2}$ = 0.12. Interestingly, this time, the truth effect (i.e., the effect of congruent statement repetition) was even larger than the source effect, as indicated by a comparison of effect-sizes (congruent repetition: ${\eta }_{\mathrm{p}}^{2}$ = 0.29; source: ${\eta }_{\mathrm{p}}^{2}$ = 0.18). We again did not find an interaction between source and repetition, *F* < 1 (see Fig. 5).

#### Linear mixed model

As for Experiment 3, we ran a linear mixed-model analysis to explore whether the above findings replicate when predicting truth judgments as a function of source trustworthiness ratings (instead of real sources vs. fake sources). The model included the fixed factors rated source trustworthiness (as a continuous variable), statement repetition (congruent vs. incongruent vs. no repetition), and their interaction as well as random intercepts for participants and statements. In line with the ANOVA findings, truth judgments were significantly affected by source trustworthiness, *F*(1, 4299.4) = 114.72, *p* < 0.001, and by statement repetition, *F*(2, 4133.3) = 127.88, *p* < 0.001. Again, there was no significant source by repetition interaction, *F* < 1.

### Discussion

Once again, we found significant effects of source credibility and statement repetition on the perceived truth of statements, but no interaction between the two factors. This was the case even though the experiment contained semantically incongruent statement repetitions in addition to congruent repetitions. In accordance with previous research, congruent repetition led to a truth effect whereas incongruent repetition led to an illusion of falseness. That is, statements that diverged from previously presented statements were perceived as less true than unfamiliar statements although participants had no factual knowledge of their validity. Moreover, source credibility did not moderate this illusion of falseness. This means that participants did not increase their attention to source information when encountering information that was incongruent to earlier encountered information. Hence, even for incongruent statements allegedly presented by real news sources, there was still a decrease in rated truth (both in comparison to congruently repeated statements and to new statements). To our knowledge, this is the first demonstration of the illusion of falseness in a social media news context. We will outline the real-world implications of this finding in the general discussion section.

## General discussion

The aim of this study was to gain a better understanding of the mechanisms that contribute to people’s belief in (fake) news, with a particular focus on social media. For this reason, we investigated effects of the following variables that, according to previous research, demonstrably affect truth judgments when studied in isolation: source credibility, statement repetition, and non-probative pictures. As these variables typically co-occur in the context of social media, we were interested in their effects when being jointly manipulated.

### Summary and interpretation of results

In a series of four experiments, we found reliable effects of source credibility and repeated exposure on participants’ truth judgments. However, we did not find significant effects of truthiness—the phenomenon that non-probative pictures enhance people’s truth judgments. Given the support for a basic truthiness effect in the literature (e.g., Fenn et al. 2013; Newman et al. 2012, 2020), it is surprising that it did not replicate in our experiments. At present, we can only speculate why the effect did not occur. It is possible that truthiness is restricted to contexts in which the presence of a picture (and its absence, respectively) is the only available cue for a statement’s truth. As the effect is generally smaller than effects of source credibility and repetition, the other effects might have simply overshadowed truthiness. In line with this idea, there were at least descriptive trends in all experiments that included a picture condition for truth judgments to be higher in the picture compared to the no-picture condition. Alternatively, the failed replication of truthiness could also be based on our selection of pictures for which we had taken great care to ensure that they are non-probative. A truthiness study with our materials, but without a source and repetition manipulation would allow testing the above explanations against each other. Irrespective of which explanation is correct, however, our findings suggest that in the context of social media, pictures play a negligible role with regard to people’s belief in (fake) news, at least if the pictures are non-probative. The effects of seemingly probative pictures (either for or against a statement), in contrast, remain an interesting topic for future research.

Unlike non-probative pictures, source credibility and repeated exposure turned out to be reliable determinants of judged truth. Truth judgments were higher for statements presented with credible sources than non-credible sources and statements presented without source information. Moreover, congruent statement repetition increased perceived truth whereas semantically incongruent repetition decreased perceived truth. Importantly, the effects of source credibility and repetition were additive, in line with previous work by Unkelbach and Greifeneder (2018), who had also found additive effects of declarative and experiential cues on judged truth. When comparing the size of the source effect with the size of the truth effect—the credibility enhancing effect of congruent statement repetition—the former was larger in Experiments 2 and 3 whereas the latter was larger in Experiment 4. Hence, the weighting of source credibility and fluency seems to be context-dependent. It is possible that differences in the experimental designs affected cue salience and thus resulted in a stronger weighting of source credibility in Experiments 2 and 3. For example, the lack of source information in the no-source condition of these experiments may have particularly attracted participants’ attention to the source cue.

In addition to the varying size of the source credibility effect, the pattern of this effect also differed between experiments. In Experiment 1 and 2, we manipulated source credibility by source expertise, i.e., statements allegedly stemmed from an expert source, a lay source, or appeared without source information. In the other two experiments, by contrast, we manipulated source credibility by source trustworthiness. In Experiment 3, for example, alleged news headlines were either presented by a trustworthy news source, a fake source (i.e., a made-up source that looks like a real news source but actually does not exist), or appeared without source information. Interestingly, although expert sources and trustworthy news sources both increased the perceived truth of statements compared to the no-source condition, we observed different results for the lay sources and fake sources. Statements in the lay-source condition were rated as less true than statements without source information, which suggests that participants discounted the information provided by laypersons. In contrast, this did not happen for the fake sources. Headlines in the fake-source condition received similar truth ratings as headlines presented without source information. Based on this discrepancy, it would be interesting to investigate whether information presented by lay sources is perceived as less credible than information from unknown news sources. As this goes beyond the scope of our article, it remains an interesting point for future studies.

### Implications for the perceived truth of social media news

Based on Lazer et al.’s (2018) definition of fake news cited at the beginning of this article, an important indicator for the veracity of encountered information is its source. Thus, it is good news that source credibility proved to be a reliable determinant of judged truth in our experiments, replicating earlier findings (see Wilson and Sherrell 1993). In addition, however, participants were susceptible to the truth effect, i.e., the credibility-enhancing effect of statement repetition (see Dechêne et al. 2010). In contexts in which source information and previous exposures are ecologically valid cues for a statement’s truth (e.g., in educational contexts), it makes perfect sense that people rely on both cues when forming truth judgments (for source information, von der Mühlen et al. 2016; for repetition, Reber and Unkelbach 2010). In the context of social media, however, statement familiarity is not a valid cue for truth, as fake news may even spread faster and more broadly than real news (Vosoughi et al. 2018).

Given the additive nature of the source credibility effect and the repetition effect, participants always provided the highest truth judgments for congruently repeated information presented by credible sources. Truth judgments in the other conditions, by contrast, varied depending on participants’ cue weighting. It is of particular interest that in Experiment 4, in which all statements appeared with source information, the truth effect for congruently repeated statements was even stronger than the source credibility effect. Consequently, participants judged congruently repeated headlines presented by dubious sources as more likely true than new or incongruently repeated headlines presented by trustworthy news sources, *t*(79) = 2.32, *p* = 0.023, *d*_*z*_ = 0.26, and *t*(79) = 3.66, *p* < 0.001, *d*_*z*_ = 0.41, respectively (see Fig. 5). Moreover, headlines that were incongruent with previously read information produced an illusion of falseness within each of the source conditions; these headlines were rated as less true than new headlines. Taken together, these findings imply that the order of information processing influences people’s evaluations of (fake) news. People tend to believe information they have encountered before and to distrust information that is inconsistent with previously encountered information. Hence, whatever information comes first has a higher chance of being believed.

In contexts in which sources are particularly distinctive (Experiments 2 and 3), however, source credibility seems to have a stronger impact on judged truth than repetition does. We therefore believe that a promising intervention to combat fake news is to draw people’s attention to source information. In line with Rapp and Salovich (2018), we suggest that this could be promoted by specific educational programs that train people how to distinguish trustworthy from untrustworthy sources and how to use source cues to evaluate und compartmentalize information. However, as explicit knowledge about source credibility is not necessarily taken into account during information processing (Sparks and Rapp 2011), context-specific interventions could additionally help to increase people’s attentiveness to source credibility. For instance, similar to the labelling of “trusted shops” on the Internet, social media platforms could tag “trusted sources” by special badges. Of course, the accreditation as a trusted source would have to come from acknowledged and politically independent institutions. Likewise, sources that do not perform well in a fact check could be tagged as “dubious sources.” In fact, Rapp (2016) assumes that “tagging should be particularly effective when it marks who or what is *not* reliable” (p. 284). It remains to be seen, however, whether the source-tagging strategy we propose is a more promising approach to combat fake news on social media than tagging of individual postings (see Pennycook et al. 2018).

### Directions for future research

Although our experiments provide clear evidence that source credibility and repeated exposure have strong, additive effects on judged truth, follow-up studies are warranted. Such studies might include different time intervals between repetitions of statements, as the length of retention interval has been shown to be a key factor in the illusion of falseness (Garcia-Marques et al. 2015). By presenting source information in the exposure phase, future studies could additionally explore the role of source memory and source variability in truth judgments. Going beyond source credibility, repetition, and pictures, future studies may look into other criteria for judged truth. Schwarz (2018) identified five such criteria, all of which could be assessed retrieving declarative knowledge or metacognitive experiences: (1) is information compatible with previous knowledge; (2) is it internally consistent and plausible; (3) supported by evidence; (4) accepted by others; and (5) offered by a credible source. The current study addressed two of these criteria, source credibility in all experiments and internal consistency in Experiment 4. In future studies, it will be important not only to examine the effects of single criteria on truth judgments but to assess the relative importance of individual factors by combining them in ecologically valid studies. Finally, given the fact that we did not investigate factual but simulated social media postings, our findings should be validated under more realistic conditions. In this regard, it would also be interesting to investigate more complex materials such as whole news stories (e.g., Polage 2012) and to explore further potential determinants of judged truth in the context of social media news such as “likes” and user comments (e.g., Heinbach et al. 2018; Lewandowsky et al. 2019).

## Conclusion

In summary, we found strong, additive effects of source credibility and repetition on the perceived truth of statements and simulated social media postings, but no effects of non-probative pictures. These results provide support for the theoretical assumption that people simultaneously rely on declarative and experiential cues when forming truth judgments. Moreover, as the weighting of cues seems to depend on their salience, making credible and non-credible sources more distinctive could be a promising intervention to combat fake news on social media. Our findings thus bear relevance not only from a theoretical, but also from an applied perspective.

## Data Availability

The materials and all datasets generated and analyzed during the current study are publicly available online at the Open Science Framework (OSF; https://osf.io/bnqgs/).

## Footnotes

1 For a detailed explanation of why fluency is an ecologically valid cue for truth, see Reber and Unkelbach (2010).

2 Because G*Power does not include a built-in module to directly run power analyses for interactions between repeated-measure factors, we used the Generic *F* test module for these power calculations.

3 By mistake, the picture was omitted in one statement with an expert source that should have had one, leading to imbalance in one statement. We coded this statement accordingly in the statistical analysis.

4 By mistake, there was again an imbalance of one statement. Instead of being presented without a source the item appeared with an expert source in the picture condition. We coded this statement accordingly in the statistical analysis.

5 We did not exclude non-native participants for the following reasons. A truth effect study by Nadarevic et al. (2018) found that truth judgments do not differ between native and foreign-language statements if the lag between statement repetitions is relatively short as in the present study. Moreover, excluding non-native participants did not change the general pattern and significance of results.
